# Impact of Maternal Diabetes on Epigenetic Modifications Leading to Diseases in the Offspring

**DOI:** 10.1155/2012/538474

**Published:** 2012-11-22

**Authors:** Nikolaos Vrachnis, Nikolaos Antonakopoulos, Zoe Iliodromiti, Konstantinos Dafopoulos, Charalambos Siristatidis, Kalliopi I. Pappa, Efthymios Deligeoroglou, Nicolaos Vitoratos

**Affiliations:** ^1^2nd Department of Obstetrics and Gynecology, Aretaieion Hospital, University of Athens Medical School, 11528 Athens, Greece; ^2^1st Department of Obstetrics and Gynecology, Alexandra Hospital, School of Medicine, University of Athens, 11528 Athens, Greece; ^3^Neonatal Unit, 2nd Department of Obstetrics and Gynecology, Aretaieion Hospital, University of Athens Medical School, 11528 Athens, Greece; ^4^Department of Obstetrics and Gynecology, School of Medicine, University of Thessaly, 41334 Larissa, Greece; ^5^3rd Department of Obstetrics and Gynecology, Attiko Hospital, University of Athens, 12462 Athens, Greece

## Abstract

Gestational diabetes, occurring during the hyperglycemic period of pregnancy in maternal life, is a pathologic state that increases the incidence of complications in both mother and fetus. Offspring thus exposed to an adverse fetal and early postnatal environment may manifest increased susceptibility to a number of chronic diseases later in life. Compelling evidence for the role of epigenetic transmission in these complications has come from comparison of siblings born before and after the development of maternal diabetes, exposure to this intrauterine diabetic environment being shown to cause alterations in fetal growth patterns which predispose these infants to developing overweight and obesity later in life. Diabetes of the offspring is also mainly the consequence of exposure to the diabetic intrauterine environment, in addition to genetic susceptibility. Since obesity and diabetes are known to increase the risk of cardiovascular disease, cardiovascular sequelae in the offspring of diabetic mothers are virtually inevitable. Research data also suggest that exposure to a diabetic intrauterine environment during pregnancy is associated with an increase in dyslipidemia, subclinical vascular inflammation, and endothelial dysfunction processes in the offspring, all of which are linked with development of cardiovascular disease later in life. The main underlying mechanisms involve persistent hyperglycemia hyperinsulinemia and leptin resistance.

## 1. Introduction

The prevalence of obesity and type 2 diabetes amongst all age groups has, in the present time, reached alarming levels. As a result, more and more women of child-bearing age are either obese and/or diabetic during pregnancy. Pregnancy is a progressively hyperglycemic period, maternal hyperglycemia being necessary for the nutritional needs of the growing fetus [1]. However, maternal diabetes is a pathologic state that increases the incidence of complications in both the mother and the fetus [2], while it also exposes the women affected to higher risk for metabolic syndrome, subsequent type 2 diabetes, and cardiovascular disease later in life [3]. Long-term consequences for the offspring are also significant. Adipokines and inflammatory mediators are the main factors causing chronic subclinical inflammation which leads to insulin resistance and abnormality in glucose metabolism [1, 3], while there is additionally increasing evidence that exposure to an adverse fetal and/or early postnatal environment may increase susceptibility to a number of chronic diseases later in the life of the offspring. There is thus great interest in identifying the exact mechanisms by which maternal excess glucose may lead to diseases in the offspring later in life for the development of strategies to prevent this destructive cycle of metabolic dysfunction through generations.

Depending on the diagnostic and screening criteria, it has been observed that the prevalence of gestational diabetes ranges from 1.3% to 19.9% [4]. The recently established criteria use a 75 g oral glucose tolerance test (OGTT) without prior glucose challenge and diagnose gestational diabetes when the fasting glucose is ≥5.1 mmol/L and/or when the 1 h postload glucose is ≥10.0 mmol/L and/or when the 2 h postload glucose is ≥8.5 mmol/L [5]. Previousgestational diabetes, advanced maternal age, and obesity have the highest impact ongestational diabetesrisk. Racial/ethnic origin and family history of type 2diabeteshave a significant though moderate impact (except for type 2diabetesin siblings). Several nontraditionalfactors have recently been characterized, these being either physiological (low birthweight and short maternal height) or pathological (polycystic ovaries). The combination of the multiplicity ofrisk factorsand their interactions results in a low reliability ofriskprediction on an individual basis [6]. Traditional management includes diet, exercise, and short- and intermediate-acting insulin regimens. Meanwhile, use of metformin and glyburide is still controversial, but evidence substantiating their safety and efficacy is accumulating. Finally, postpartum screening with a glucose tolerance test rather than a fasting blood glucose level should be performed 6 weeks after delivery [7].

## 2. Offspring Diseases due to Hyperglycemic Intrauterine Environment

### 2.1. Offspring Growth and Adiposity

Offspring of diabetic mothers display excess fetal growth resulting in macrosomic and large for gestational age (LGA) infants [8], hence contributing to the increased risk for cesarean section or traumatic birth [9]. This excess fetal growth is caused by increased nutrients availability from the mother to the developing fetus through the placenta. Maternal serum glucose, which is the main excess nutrient in these circumstances, freely crosses the placenta, while maternal insulin does not. As a result of the thus induced fetal hyperglycemia, the fetal pancreas, although immature, is capable of producing increased levels of insulin, which in turn acts as a growth hormone and promotes growth and adiposity in the fetus [10]. The degree of hyperglycemia seems to determine the metabolic effect on the neonate [11]. Additionally to glucose excess, alterations in the delivery of amino acids and upregulation of placental transport systems also contribute to increased fetal growth [9, 12]. Notably, exposure to this intrauterine diabetic environment causes alterations in fetal growth patterns which predispose these infants to being overweight and obese also later in life, even in the absence of macrosomia at birth [13]. Insulin seems to play a key role, since amniotic fluid insulin levels during the third trimester correlate independently with infants' obesity [14].

### 2.2. Offspring Glucose Tolerance Disturbances

Many studies have shown that offspring of diabetic mothers have a statistically higher incidence of impaired glucose tolerance (IGT), this constituting a well known prediabetic state [15]. In the case of gestational diabetes, offspring have been shown to display reduced insulin secretion, while in the case of pre-existing diabetes, offspring have been shown to exhibit heightened insulin resistance, this possibly indicating a small difference in the underlying mechanisms [16]. Past studies also demonstrated that in all age groups of offspring who were exposed to hyperglycemia in utero, the incidence of diabetes was higher compared with the offspring of nondiabetic mothers, even though some of the latter would still develop diabetes in the future [17]. Therefore, offspring diabetes is mainly the consequence of exposure to a diabetic intrauterine environment, in conjunction with genetic susceptibility. 

### 2.3. Offspring Cardiovascular Abnormalities

**Figure 1:**
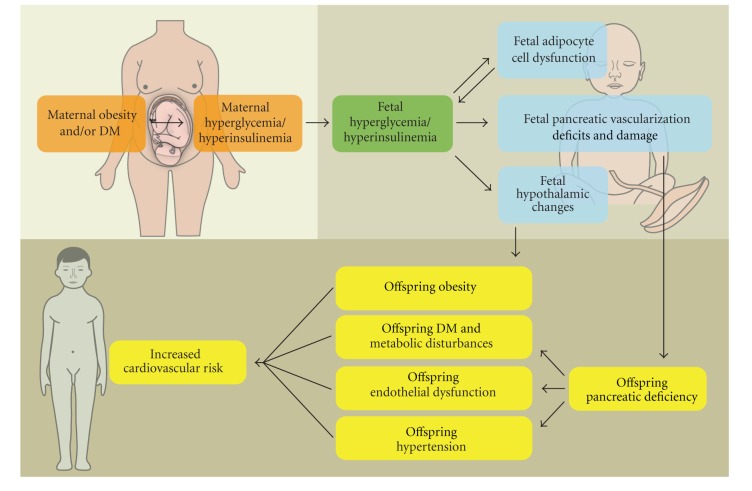
Maternal hyperglycemia and hyperinsulinemia and, consequently, fetal hyperglycemia and hyperinsulinemia alter the function of every stage of fetal metabolism, including the hypothalamus, the pancreas, and adipose tissue. These metabolic disturbances pass through the offspring and, eventually, could increase cardiovascular risk for the maturing young adult.

The main issue is whether these metabolic abnormalities of the offspring can augment their risk for cardiovascular diseases later in life, since such a finding would prompt closer glycemic control of their diabetic mothers and, possibly, closer health surveillance of the offspring themselves as a high risk population. However, few human studies have examined the effect of a diabetic intrauterine environment on cardiovascular risk factors of the offspring (Figure 1). Nevertheless, since obesity and diabetes are known to increase the risk of cardiovascular disease, the assumption is that cardiovascular consequences will arise in the offspring of diabetic mothers [18]. 

Evidence of cardiovascular changes in pregnancies complicated by diabetes is already apparent during the third trimester of in utero life. The fetal heart shows reduced ventricular contractility compared with pregnancies not complicated by diabetes, even if the latter were complicated by hypertensive disease [19]. These findings suggest that the diabetic intrauterine environment induces biochemical alterations in the cardiovascular system that affect its function and that these changes are distinct from those caused by other poor intrauterine environments, such as those seen in hypertensive pregnancies. 

In addition, systolic blood pressure, a well-known risk factor for cardiovascular diseases, of children born to diabetic mothers was significantly higher than that of those born to nondiabetic mothers [14, 20].

Research data also suggest that exposure to a diabetic intrauterine environment during pregnancy is associated with an increase in dyslipidemia, subclinical vascular inflammation, and endothelial dysfunction processes in the offspring, all of which are linked with development of cardiovascular disease later in life. Dyslipidemia is expressed by increased total and LDL cholesterol. The vascular inflammation and endothelial dysfunction markers examined were plasminogen activator inhibitor-1 (PAI-1), vascular adhesion molecule-1 (VCAM), intercellular adhesion molecule-1 (ICAM), E-selectin, insulin-like growth factor-1 (IGF-1), and others [21]. Thus, women with gestational diabetes and their fetuses demonstrate alterations in markers of eNOS uncoupling, oxidative stress, and endothelial dysfunction, and these changes correlate with the levels of hyperglycemia [22].

## 3. Epigenetic Modifications

Although diabetes type 2 is a disease with a known genetic component and is usually associated with a positive family history, this is not always the case. In fact, the current increasing incidence of the disease, which has risen to almost epidemic proportions, reveals that there must be a potent environmental component contributing to the disease as well. Many postnatal risk factors have been studied, like obesity, poor physical activity, and inappropriate diet. Nevertheless, prenatal exposure to a diabetic intrauterine environment seems to contribute to the development of the disease in the offspring later in life. Among females, it additionally contributes to the development of gestational diabetes, thus promoting a positive feedback loop for this disease. Animal models reveal that this metabolic imprinting can be transmitted across generations [23, 24].

Children who were exposed to a diabetic intrauterine environment during pregnancy are more likely to be obese [25], and there is a higher incidence of diabetes later in life among them than that is genetically expected, by almost 40% [26]. Adjustment for maternal weight does not explain the excess risk of the offspring, thereby supporting the hypothesis that nutrient-mediated developmental abnormalities in utero contribute independently to the development of obesity and diabetes in the offspring of diabetic mothers [13, 25].

Moreover, an excess of maternal to paternal transmission of diabetes has been widely reported, suggesting an epigenetic transmission [27, 28]. In other words, the extent to which maternal transmission exceeds paternal transmission can only be attributed to the intrauterine exposure to diabetes.

However, the strongest evidence for the role of epigenetic transmission has come from the comparison of siblings born before and after the development of maternal diabetes. Since siblings have the same risk of carrying the “diabetic” genes of their mothers, the excess risk that was observed in those born after the development of diabetes in their mothers can only be attributed to the hyperglycemic intrauterine environment [29].

Early epigenetic effects, such as those of an intrauterine hyperglycemic environment, play a significant role not only in the development of disease but also in its course. This is shown by studies in which offspring of mothers with diabetes before pregnancy developed diabetes at a younger age than those of mothers without diabetes who developed diabetes later after labor [30].

Finally, postnatal nutritional status is another epigenetic surfactant that influences the risk of diabetes in the offspring of diabetic mothers, with breastfeeding being protective [31]. We could thus reasonably suggest that the risk of diabetes is a combination of early prenatal and postnatal factors that act on a genetic basis and can trigger the disease sooner or even de novo. 

## 4. Underlying Mechanisms

It is by now well established that insulin promotes storage of excess nutrition and fat mass, while at the same time leptin production from adipocytes acts as a safe mode negative loop mechanism that suppresses further insulin secretion. Interestingly, these two parameters, insulin and leptin, seem to play key roles in the metabolic disturbance [32]. 

There is evidence that hyperinsulinism can cause metabolic changes in the offspring of normoglycemic mothers. This evidence has come from animal experiments and reveals that hyperinsulinism rather than hyperglycemia could be the main surfactant acting on the offspring of diabetic mothers [33]. 

**Figure 2:**
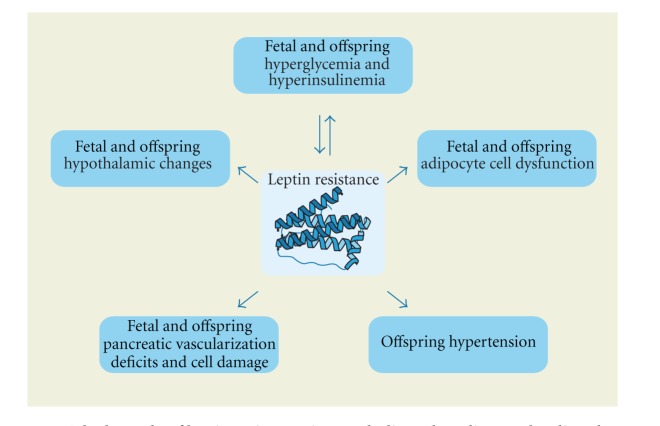
The key role of leptin resistance in metabolic and cardiovascular disturbances.

On the other hand, cord blood levels of leptin are also higher in the offspring of diabetic mothers than nondiabetic [34]. It cannot thus be hypothesized that a deficiency of leptin production in the case of diabetes has a role in the pathology of the offspring. Even more significantly, there is no connection between leptin and IGF-1 [35], though neither does the increase of leptin seem to act prophylactically for the offspring. What could hence be hypothesized is a type of leptin resistance in the case of neonates of diabetic mothers as a result of persistent hyperglycemia and hyperinsulinemia, just as insulin resistance is a progenitor of diabetic pathology. This leptin resistance might be the result of dysfunction of adipocytes or pancreatic cells and could be the cause of further hyperinsulinemia and a progressive positive loop mechanism (Figure 2).

However, elevated leptin concentrations during diabetic pregnancy may be due to its secretion by adipocytes in the presence of elevated estrogen and also by placenta. In gestational diabetes, adipose tissue secretes low adiponectin (an anti-inflammatory and positive stimulator of insulin sensitizing) and high TNF-*α* and IL-6, which contribute to the inflammatory state and insulin resistance present in diabetic pregnancy as well as in macrosomia [36]. Leptin, being proinflammatory, is produced in abundance by adipose tissue during diabetic pregnancy and has been implicated in the pathogenesis of weight gain in macrosomic babies. Leptin may exert its effects by interacting with neuropeptide-Y in the hypothalamus, while the intrauterine hyperglycemia may act on the fetal hypothalamus and create what has been termed a “metabolic memory” which programs for development of obesity and metabolic syndrome in the offspring during adulthood [37].

As this condition goes on, further damage of pancreatic cells, as a result of severe hyperglycemia, is liable to cause defective insulin secretion and could be the starting point for initiation of glucose tolerance disturbances [38]. In addition, some data exist concerning intrauterine pancreatic vascularization deficits through alterations in factors like vascular endothelial growth factor (VEGF) and its receptors and intrauterine pancreatic innervation disturbances in diabetic pregnancies [38, 39].

## 5. Conclusions

The present-day diabetes epidemic incurs enormous costs, especially when one takes into account the adverse consequences wrought upon the future lives of both mother and offspring. Reducing obesity and type 2 diabetes should therefore be a primary goal of public health organizations and clinicians. Meanwhile, in the case of diabetic pregnancies, it is necessary to bear in mind that offspring metabolic dysfunction is a composite result of genetic and epigenetic factors, the latter also involving alterations in placental transport, endocrine molecules, like insulin and leptin, and inflammatory markers. Thus, with regard to the clinical management of women with diabetes during pregnancy, there may be the need to focus not only on achieving tight control of maternal blood glucose levels, but also on additional dietary and/or other changes, such as encouragement of breastfeeding. In summary, there is clearly an urgent need for the accumulation of specialized knowledge as to the most effective strategies to deal with metabolic disturbances and risk for chronic diseases in the offspring of diabetic mothers as a result of in utero exposure to diabetes.
